# Mortality and Cardiovascular Events in Patients With Chronic Kidney Disease and Sleep Apnea Syndrome

**DOI:** 10.3389/fmed.2022.899359

**Published:** 2022-05-31

**Authors:** Yu Watanabe, Akihito Tanaka, Kazuhiro Furuhashi, Shoji Saito, Shoichi Maruyama

**Affiliations:** ^1^Department of Nephrology, Nagoya University Graduate School of Medicine, Nagoya, Japan; ^2^Department of Nephrology, Nagoya University Hospital, Nagoya, Japan

**Keywords:** sleep apnea syndrome, chronic kidney disease, mortality, CPAP, SAS

## Abstract

**Background:**

The incidence of sleep apnea syndrome (SAS) is reported to be markedly high in patients with chronic kidney disease (CKD). Therefore, it is extremely important to know whether SAS affects prognosis in patients with CKD. Further, it is imperative to understand the prognostic impact of home continuous positive airway pressure (CPAP) therapy, which is one of the most common treatments for SAS.

**Materials and Methods:**

We used a clinical database to identify patients with CKD using diagnosis codes. We included patients with CKD aged 20 years or more, not on renal replacement therapy, with a known change in renal function for at least 1 year. The propensity score was used to compare event rates for patients with SAS and those without SAS. In addition, the prognostic impact of CPAP therapy was investigated. The primary outcome is a composite of death, initiation of renal replacement therapy, hospitalization for heart failure, ischemic heart disease, and cerebrovascular disease.

**Results:**

From the database, 31,294 patients with CKD without SAS and 1,026 with SAS were found to be eligible. Of these, 419 (41%) patients with SAS and 10,713 (34%) patients without SAS (*P* < 0.01) reached the primary outcome. After adjustment with the propensity score, the SAS group was found to have a similarly poor prognosis (*P* < 0.01): the hazard ratio for the primary outcome was 1.26 (95% CI, 1.08–1.45, *P* < 0.01) in the group with SAS compared with the group without SAS. Conversely, in patients with SAS and using CPAP, the hazard ratio was lower and did not differ significantly (HR 0.96, 95% CI: 0.76–1.22, *P* = 0.76).

**Conclusion:**

In patients with CKD and SAS, the risk of death and cardiovascular disease is high. In addition, patients treated with CPAP may have improved life expectancy.

## Introduction

Sleep apnea syndrome (SAS) is known to be associated with hypertension, cardiovascular events, and death (1, 2). In patients with chronic kidney disease (CKD), prevalence of SAS is reported to be markedly high (3–5). Therefore, it is extremely important to know whether SAS affects prognosis in CKD patients. It is important to understand the prognostic impact of home continuous positive airway pressure (CPAP) therapy, which is one of the most common treatments for SAS.

We have previously reported that fluid retention in CKD (6) and arterial stiffness are associated with SAS (7). Furthermore, several reports have indicated that the prognosis is worse in OSAS patients with CKD (8). However, it is not clear whether SAS affects prognosis in patients with CKD, and if so, to what extent. In addition, the efficacy of home CPAP therapy has been shown in randomized controlled trials to significantly reduce events in patients in the CPAP group who had better use of CPAP in a *post-hoc* analysis, although the primary endpoint did not show a significant difference (9, 10). However, the prognostic value of CPAP in patients with CKD is unknown.

In this study, we used a clinical database to compare the incidence of cardiovascular events in CKD patients with and without SAS. In addition, we investigated the prognostic impact of CPAP treatment.

## Materials and Methods

### Data Source

We obtained data from Medical Data Vision Co., Ltd (MDV), which was one of the largest hospital claims registries in Japan. Data from MDV is built from hospital claims data, including individual procedures, prescriptions, examinations, surgery, hospitalization and laboratory data. MDV uses International Classification of Diseases, Tenth Revision (ICD-10) coding. Data collection started in April 2008. As of August 2021, MDV had collected 36,690,000 individual patient records from 449 hospitals across Japan. We identified and selected a group of patients defined by disease codes (N18, N03, and N11) that we considered to correspond to patients with CKD. We obtained data for 924,238 patients.

### Study Design and Patient Selection

Patients with CKD aged 20 years or older with a history of at least two serum creatinine measurements during the observation period and a known renal function trend of at least 1 year were included. Patients who were already receiving renal replacement therapy (hemodialysis, peritoneal dialysis, or renal transplantation) at the start of observation were excluded. Patients whose renal replacement therapy induction date was not known in the database, but who were prescribed dialysis were excluded as maintenance dialysis patients. The diagnosis of sleep apnea was determined by whether SAS (G473) was registered as a medical condition. The date of diagnosis was defined as the date when sleep apnea was diagnosed for the first time.

The presence or absence of CPAP treatment was collected from data on treatment costs (receipt code 1114041310), and patients with a history of CPAP prescription during the observation period were defined as CPAP-treated patients.

### Covariates and Clinical Outcomes

Known cardiovascular comorbidities, including hypertension, diabetes mellitus, heart failure, atrial fibrillation, and atrial flutter, were defined according to ICD-10 codes (Supplementary Table 1). Each disease was counted as a comorbidity if it was named within the observation period. The primary outcome is a composite of death, initiation of renal replacement therapy (hemodialysis, peritoneal dialysis, renal transplantation), hospitalization for heart failure, hospitalization for ischemic heart disease, and hospitalization for cerebrovascular disease. The observation period for non-SAS patients was defined as the time from the initial visit, while the observation period for SAS patients was defined as the date of diagnosis.

### Statistical Analysis

Baseline characteristics were represented descriptively and tested using the unpaired Student’s *t*-test or χ^2^ test according to the types of data. Survival rate was represented graphically using the Kaplan–Meier method and tested by Log Rank test. A value of *p* < 0.05 was considered significant statistically.

We used propensity score matching to match the baseline characteristics. The propensity score was calculated by age, sex, past medical history of hypertension, diabetes, heart failure, arterial fibrillation, arterial flutter, and laboratory data [estimated glomerular filtration rate (eGFR), hemoglobin and Na]. The level of eGFR was calculated using the Japanese formula (11).

We used R software (R Foundation for Statistical Computing, Vienna, Austria)^1^ to conduct all statistical analyses in this study. For propensity score matching, the R package MatchIt (1:1 matching with the nearest neighbor with caliper matching, caliper = 0.1) was used for the calculation (12).

### Ethics

Informed consent was waived because patient records were anonymized and deidentified. All deidentified data was used in accordance with local regulations.

This study was approved by the Ethics Committee of the Institutional Review Board of Nagoya University Hospital (approval number 2021-0350), and was performed in adherence to the Declaration of Helsinki.

## Results

### Baseline Characteristics

Table 1 shows the characteristics of the SAS and non-SAS patients before propensity score matching. The number of patients with SAS who received CPAP therapy was 370 (36%). Because of the large difference in patient backgrounds, we decided to perform propensity score matching.

**TABLE 1:** Baseline characteristics of patients.

Characteristic	Patients without SAS, *N* = 31,294	Patients with SAS, *N* = 1,026	*p*-value
Male, *n* (%)	19,000 (61%)	805 (78%)	< 0.01
Age (y), mean ± SD	74 (12)	70 (12)	< 0.01
CPAP, *n* (%)	1 (<0.1%)	370 (36%)	< 0.01
Past history			
HT, *n* (%)	19,189 (61%)	925 (90%)	< 0.01
DM, *n* (%)	12,140 (39%)	675 (66%)	< 0.01
HF, *n* (%)	7,618 (24%)	647 (63%)	< 0.01
Afor AFL, *n* (%)	3,305 (11%)	328 (32%)	< 0.01
Laboratory data			
Hb (g/dL), mean ± SD	12.27 (2.19)	12.60 (2.32)	< 0.01
TP (mg/dL), mean ± SD	6.98 (0.67)	6.84 (0.69)	< 0.01
Alb (mg/dL), mean ± SD	3.88 (0.54)	3.77 (0.57)	< 0.01
Cre (mg/dL), mean ± SD	1.61 (1.08)	1.77 (1.26)	< 0.01
eGFR (mL/min/1.73 m^2^), mean ± SD	37 (14)	37 (14)	0.27
KDIGO grade			0.31
G3a, *n* (%)	10,695 (34%)	323 (31%)	
G3b, *n* (%)	11,196 (36%)	387 (38%)	
G4, *n* (%)	7,304 (23%)	242 (24%)	
G5, *n* (%)	2,099 (6.7%)	74 (7.2%)	
BUN (mg/dL), mean ± SD	27 (14)	28 (14)	0.01
Na (mEq/L), mean ± SD	140.3 (3.4)	140.5 (3.2)	0.49
K (mEq/L), mean ± SD	4.47 (0.60)	4.40 (0.55)	< 0.01
Cl (mEq/L), mean ± SD	105.3 (4.3)	105.0 (4.2)	< 0.01
Primary outcome (composite), *n* (%)	10,713 (34%)	419 (41%)	< 0.01
eGFR decline per year (min/min/1.73 m^2^), mean ± SD	1.5 (8.5)	2.1 (4.8)	0.03

*CPAP, continuous positive airway pressure therapy; HT, hypertension; DM, diabetes mellitus; HF, heart failure; Af, atrial fibrillation; AFL, atrial flutter; Hb, hemoglobin; TP, total protein; Alb, albumin; Cre, creatinine; eGFR, estimated glomerular filtration rate; BUN, blood urea nitrogen; Na, sodium; K, potassium; Cl, chloride.*

Table 2 shows the characteristics of SAS and non-SAS patients after propensity score matching; the levels of albumin and potassium were significantly lower in patients with SAS than those without SAS. However, the clinical significance of low potassium is uncertain.

**TABLE 2:** Baseline characteristics of patients after propensity score matching.

Characteristic	Patients without SAS, *N* = 940	Patients with SAS, *N* = 940	*p*-value
Male, *n* (%)	727 (77%)	732 (78%)	0.78
Age (y), mean ± SD	70 (13)	71 (12)	0.33
CPAP, *n* (%)	0 (0%)	330 (35%)	<0.01
Past history			
HT, *n* (%)	856 (91%)	849 (90%)	0.58
DM, *n* (%)	627 (67%)	619 (66%)	0.70
HF, *n* (%)	606 (64%)	617 (66%)	0.59
Af or AFL, *n* (%)	279 (30%)	304 (32%)	0.21
Laboratory data			
Hb (g/dL), mean ± SD	12.58 (2.32)	12.55 (2.31)	0.53
TP (mg/dL), mean ± SD	6.94 (0.69)	6.83 (0.69)	<0.01
Alb (mg/dL), mean ± SD	3.87 (0.56)	3.76 (0.57)	<0.01
Cre (mg/dL), mean ± SD	1.74 (1.07)	1.80 (1.29)	0.84
eGFR (mL/min/1.73 m^2^), mean ± SD	36 (13)	36 (14)	0.99
KDIGO grade			0.91
G3a	275 (29%)	283 (30%)	
G3b	362 (39%)	353 (38%)	
G4	237 (25%)	232 (25%)	
G5	66 (7.0%)	72 (7.7%)	
BUN (mg/dL), mean ± SD	29 (15)	28 (14)	0.93
Na (mEq/L), mean ± SD	140.5 (3.1)	140.4 (3.2)	0.49
K (mEq/L), mean ± SD	4.46 (0.60)	4.40 (0.56)	<0.05
Cl (mEq/L), mean ± SD	105.3 (4.0)	105.0 (4.2)	0.26
Primary outcome (composite), *n* (%)	411 (44%)	398 (42%)	0.54
eGFR decline per year (min/min/1.73 m^2^), mean ± SD	1.7 (5.7)	2.1 (5.0)	0.28

*CPAP, continuous positive airway pressure therapy; HT, hypertension; DM, diabetes mellitus; HF, heart failure; Af, atrial fibrillation; AFL, atrial flutter; Hb, hemoglobin; TP, total protein; Alb, albumin; Cre, creatinine; eGFR, estimated glomerular filtration rate; BUN, blood urea nitrogen; Na, sodium; K, potassium; Cl, chloride.*

### Clinical Outcomes in Sleep Apnea Syndrome Patients Compared With Non-sleep Apnea Syndrome Patients

**FIGURE 1:**
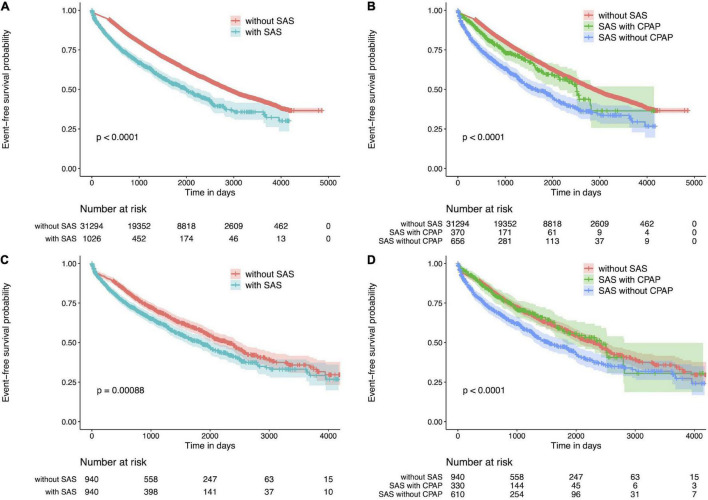
Kaplan Meier plot for CKD patients. **(A)** Kaplan Meier plot of patients with or without SAS. CKD patients with SAS have a poor prognosis. **(B)** Kaplan Meier plot for patients with or without CPAP therapy or without SAS. The event rate was lower in the group treated with CPAP. **(C)** Kaplan Meier plot for CKD patients with or without SAS after propensity score matching. CKD patients with SAS have a poor prognosis. **(D)** Kaplan Meier plot for CKD patients with or without CPAP therapy or without SAS after propensity score matching. The event rate was lower in the group treated with CPAP. CKD, chronic kidney disease; SAS, sleep apnea syndrome.

Figure 1A shows Kaplan-Meier curves for SAS and non-SAS patients before PS matching, showing that of all CKD patients, SAS patients were more likely to die, start renal replacement therapy (hemodialysis, peritoneal dialysis, renal transplantation), be hospitalized for heart failure, be hospitalized for ischemic heart disease, be hospitalized for cerebrovascular disease compared to non-SAS patients. Among SAS patients, those treated with CPAP had a lower event rate (Figure 1B). This trend was also true when PS matching was performed.

Figure 1C shows the Kaplan-Meier curves for SAS and non-SAS patients after PS matching. Compared to non-SAS patients, SAS patients with CKD were significantly more likely to die, initiate renal replacement therapy (hemodialysis, peritoneal dialysis, renal transplantation), be hospitalized for heart failure, be hospitalized for ischemic heart disease, or be hospitalized for cerebrovascular disease. In addition, event rates were lower among SAS patients treated with CPAP (Figure 1D).

**FIGURE 2:**
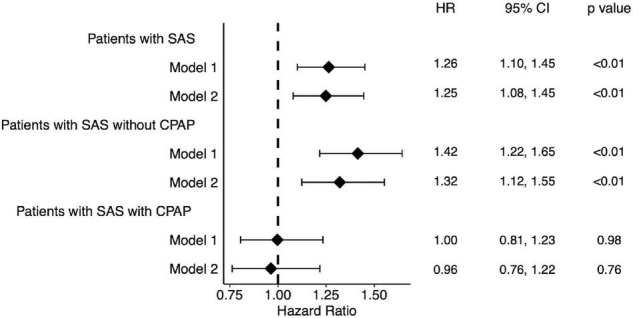
Forest plot of hazard ratio for patients with SAS. Patients with SAS showed a high hazard ratio. Among them, those treated with CPAP showed no significant difference in hazard ratios compared to the group without SAS. Model 1: without adjustment. Model 2: Adjusted by age, sex, potassium, eGFR and albumin. SAS, sleep apnea syndrome.

Figure 2 shows the hazard ratios of the primary outcome for patients with SAS and for patients with SAS who did not receive CPAP or who received CPAP compared to patients without SAS. The hazard ratio tended to be even higher. Conversely, patients with SAS who received CPAP showed no significant difference in prognosis compared to patients without SAS.

### Estimated Glomerular Filtration Rate Decline in Sleep Apnea Syndrome Patients Compared With Non-sleep Apnea Syndrome Patients

We compared the decline in eGFR per year in each group. In Table 1, we compared SAS with non-SAS patients. eGFR decline was significantly higher in SAS patients. When PS matching was performed, the same trend remained but the significant difference disappeared (Table 2). We also compared patients with and without CPAP (Supplementary Tables 2, 3) and found that eGFR decline tended to be high in patients with SAS who did not receive CPAP.

## Discussion

In the present study, we used a clinical database to investigate the rates of death, initiation of renal replacement therapy, and cardiovascular events in CKD patients with and without SAS, and found that patients with SAS had a significantly poorer outcomes. Furthermore, the study suggested that life expectancy may be improved in patients treated with CPAP.

We have previously reported that fluid retention (6), and atherosclerosis in CKD patients are associated with SAS (7). These factors would naturally be assumed to have a negative impact on life expectancy and cardiovascular events. Furthermore, it has been reported that prognosis is worse in SAS patients with comorbidities (8). In the previous report, the combination of CKD and CVD or moderate to severe chronic obstructive pulmonary disease conferred the highest risk, and CKD itself seems to have a strong negative impact on SAS. However, the extent of the impact of SAS in CKD patients has previously remained unclear. Our results are significant because they provide an answer to this question.

Furthermore, there are currently no randomized, controlled trials that have found significant differences in the efficacy of home continuous positive pressure ventilatory therapy on primary endpoints, including life expectancy and cardiovascular events. In one trial, a *post-hoc* analysis showed a significant reduction in cardiovascular events, including new onset of hypertension, among patients in the CPAP group who had better adherence to the treatment schedule. Another trial, the SAVE trial, (10) found an effect of CPAP treatment on the occurrence of cerebrovascular events in a comparison of two groups matched only for patients with good CPAP use. Although no significant differences were found in the primary endpoint, better CPAP use was significantly associated with improved prognosis (13). Therefore, it was thought that the therapeutic effect of CPAP may be even more limited in CKD patients, who have more additive adverse prognostic effects. Nevertheless, CPAP treatment was effective in this study. Since this study picked up cases of CPAP use from the addition of procedures, it is possible that patients with good usage status were selected. Alternatively, the cases for which CPAP was indicated may have been a more severely ill group that was more likely to benefit from the treatment.

One of the mechanisms associated with CKD and SAS is fluid accumulation in the neck in the supine position and narrowing of the upper airway (14). Although fluid retention is detrimental to SAS, it is also detrimental to cardiovascular events such as heart failure, and it is consistent with the mechanism that CKD, when complicated by SAS, is particularly detrimental to cardiovascular events. Furthermore, CPAP may have a positive effect on fluid retention, both in SAS itself and in heart failure, possibly improving both. Therefore, it is possible that CPAP may be particularly effective, in SAS patients with CKD and a tendency for fluid retention.

As for eGFR decline per year, the was no significant difference after PS matching. It would be difficult to explain the reduction in outcome solely by the preservation of renal function. We suggest the direct effect of CPAP on cardiac events in patients with SAS who received CPAP.

The potassium levels in patients with SAS were lower than in patients with non-SAS. This may possibly be related to the increase in aldosterone action (15). But we don’t have data of the aldosterone levels and we can’t confirm that.

Despite its strengths, this study has some limitations. First, the data is from a medical database, meaning the detailed patient backgrounds are unknown. In addition, it is not possible to track treatment provided at multiple medical institutions, so it is not known whether the patients were hospitalized or treated at other hospitals.

Second, the Apnea hypopnea index, which indicates the severity of SAS, is unknown. Therefore, the severity of SAS differs between the groups that received CPAP treatment and those that did not, and it is possible that the patient background was not properly adjusted by propensity score matching.

## Conclusion

In CKD patients with SAS, the risk of death and cardiovascular disease is high. In addition, patients treated with CPAP may have improved life expectancy.

## Data Availability Statement

The data analyzed in this study is subject to the following licenses/restrictions: We obtained the data from Medical Data Vision Co., Ltd., under an agreement not to release the original database to outside parties. Requests to access these datasets should be directed to AT, tanaka17@med.nagoya-u.ac.jp.

## Ethics Statement

The studies involving human participants were reviewed and approved by the Ethics Committee of the Institutional Review Board of Nagoya University Hospital. Written informed consent for participation was not required for this study in accordance with the national legislation and the institutional requirements.

## Author Contributions

YW and AT wrote the manuscript. YW, AT, KF, SS, and SM conceptualized the research idea and study design, performed the analysis, and carried out interpretations. AT performed the data acquisition. AT and SM provided supervision or mentorship. All authors contributed important intellectual content during the writing of the manuscript and its revisions, personally accountable for their own contributions, and agreed to ensure that questions pertaining to the accuracy and integrity of any portion of the work are appropriately investigated and resolved.

## Conflict of Interest

The authors declare that the research was conducted in the absence of any commercial or financial relationships that could be construed as a potential conflict of interest.

## Publisher’s Note

All claims expressed in this article are solely those of the authors and do not necessarily represent those of their affiliated organizations, or those of the publisher, the editors and the reviewers. Any product that may be evaluated in this article, or claim that may be made by its manufacturer, is not guaranteed or endorsed by the publisher.

## Footnotes

1
http://www.R-project.org/
