# Supplementary material for: Mortality and Cardiovascular Events in Patients With Chronic Kidney Disease and Sleep Apnea Syndrome
Source: Front Med (Lausanne). 2022 May 31;9:899359. doi: 10.3389/fmed.2022.899359 (PMC9192968; doi:10.3389/fmed.2022.899359)
Supplement: Supplementary file 1 [file Table_1.docx]

Supplemental Table S1. ICD-10 code of disease

| Disease Name | ICD10 code |
| --- | --- |
| HT | I10, I11, I12, I13, I14, I15 |
| DM | E10, E11, E12, E13, E14 |
| HF | I50, I110 |
| MI | I21, I22, I23, I24 |
| Cerebrovascular Disease | I60, I61, I62, I63, I64, I65, I66, I67, I68, I69, G45 |
| Af, AFL | I48 |
| Cardiac Event | I21, I22, I23, I44, I45, I46, I47, I48, I49 |

HT: hypertension

DM: diabetes mellitus

HF: heart failure

MI: myocardial infarction

Af: atrial fibrillation

AFL: atrial flutter

Supplemental Table S2. Baseline characteristics of patients

| Characteristic | Patients without SAS,  N = 31,294 | Patients with SAS who received CPAP therapy,  N = 370 | Patients with SAS who didn’t receive CPAP therapy,  N = 656 |
| --- | --- | --- | --- |
| Male, n (%) | 19,000 (61%) | 312 (84%) | 493 (75%) |
| Age (y), mean±SD | 74 (12) | 68 (12) | 72 (12) |
| CPAP, n (%) | 1 (<0.1%) | 370 (100%) | 0 (0%) |
| Past History |  |  |  |
| HT, n (%) | 19,189 (61%) | 339 (92%) | 586 (89%) |
| DM, n (%) | 12,140 (39%) | 239 (65%) | 436 (66%) |
| HF, n (%) | 7,618 (24%) | 216 (58%) | 431 (66%) |
| Af or AFL, n (%) | 3,305 (11%) | 106 (29%) | 222 (34%) |
| Laboratory Data |  |  |  |
| Hb (g/dL), mean±SD | 12.27 (2.19) | 13.00 (2.17) | 12.38 (2.37) |
| TP (mg/dL), mean±SD | 6.98 (0.67) | 6.90 (0.67) | 6.81 (0.69) |
| Alb (mg/dL), mean±SD | 3.88 (0.54) | 3.83 (0.55) | 3.74 (0.58) |
| Cre (mg/dL), mean±SD | 1.61 (1.08) | 1.82 (1.39) | 1.74 (1.18) |
| eGFR (mL/min/1.73m^2^), mean±SD | 37 (14) | 37 (14) | 37 (14) |
| KDIGO Grade |  |  |  |
| G3a, n (%) | 10,695 (34%) | 126 (34%) | 197 (30%) |
| G3b, n (%) | 11,196 (36%) | 133 (36%) | 254 (39%) |
| G4, n (%) | 7,304 (23%) | 84 (23%) | 158 (24%) |
| G5, n (%) | 2,099 (6.7%) | 27 (7.3%) | 47 (7.2%) |
| BUN (mg/dL), mean±SD | 27 (14) | 27 (13) | 28 (14) |
| Na (mEq/L), mean±SD | 140.3 (3.4) | 140.6 (2.9) | 140.4 (3.4) |
| K (mEq/L), mean±SD | 4.47 (0.60) | 4.44 (0.52) | 4.38 (0.57) |
| Cl (mEq/L), mean±SD | 105.3 (4.3) | 105.4 (3.5) | 104.8 (4.5) |
| Primary outcome (composite), n (%) | 10,713 (34%) | 115 (31%) | 304 (46%) |
| eGFR decline per year (min/min/1.73m^2^), mean±SD | 1.5 (8.5) | 1.9 (4.5) | 2.1 (5.0) |
| CPAP: continuous positive airway pressure therapy  HT: hypertension  DM: diabetes mellitus  HF: heart failure  Af: atrial fibrillation  AFL: atrial flutter  Hb: hemoglobin  TP: total protein  Alb: albumin  Cre: creatinine  eGFR: estimated glomerular filtration rate  BUN: blood urea nitrogen  Na: sodium  K: potassium  Cl: chloride | | | |

Supplemental Table S3. Baseline characteristics of patients after propensity score matching

| Characteristic | Patients without SAS,  N = 940 | Patients with SAS who received CPAP therapy,  N = 330 | Patients with SAS who didn’t receive CPAP therapy,  N = 610 |
| --- | --- | --- | --- |
| Male, n (%) | 727 (77%) | 277 (84%) | 455 (75%) |
| Age (y), mean±SD | 70 (13) | 68 (12) | 72 (12) |
| CPAP, n (%) | 0 (0%) | 330 (100%) | 0 (0%) |
| Past History |  |  |  |
| HT, n (%) | 856 (91%) | 301 (91%) | 548 (90%) |
| DM, n (%) | 627 (67%) | 215 (65%) | 404 (66%) |
| HF, n (%) | 606 (64%) | 203 (62%) | 414 (68%) |
| Af or AFL, n (%) | 279 (30%) | 98 (30%) | 206 (34%) |
| Laboratory Data |  |  |  |
| Hb (g/dL), mean±SD | 12.58 (2.32) | 12.96 (2.18) | 12.33 (2.36) |
| TP (mg/dL), mean±SD | 6.94 (0.69) | 6.90 (0.67) | 6.80 (0.70) |
| Alb (mg/dL), mean±SD | 3.87 (0.56) | 3.82 (0.54) | 3.73 (0.58) |
| Cre (mg/dL), mean±SD | 1.74 (1.07) | 1.89 (1.45) | 1.76 (1.20) |
| eGFR (mL/min/1.73m^2^), mean±SD | 36 (13) | 36 (14) | 36 (14) |
| KDIGO Grade |  |  |  |
| G3a | 275 (29%) | 104 (32%) | 179 (29%) |
| G3b | 362 (39%) | 118 (36%) | 235 (39%) |
| G4 | 237 (25%) | 81 (25%) | 151 (25%) |
| G5 | 66 (7.0%) | 27 (8.2%) | 45 (7.4%) |
| BUN (mg/dL), mean±SD | 29 (15) | 28 (14) | 29 (14) |
| Na (mEq/L), mean±SD | 140.5 (3.1) | 140.6 (2.9) | 140.4 (3.4) |
| K (mEq/L), mean±SD | 4.46 (0.60) | 4.44 (0.52) | 4.38 (0.57) |
| Cl (mEq/L), mean±SD | 105.3 (4.0) | 105.3 (3.6) | 104.8 (4.5) |
| Primary outcome (composite), n (%) | 411 (44%) | 108 (33%) | 290 (48%) |
| eGFR decline per year (min/min/1.73m^2^), mean±SD | 1.7 (5.7) | 2.0 (4.7) | 2.2 (5.1) |
| CPAP: continuous positive airway pressure therapy  HT: hypertension  DM: diabetes mellitus  HF: heart failure  Af: atrial fibrillation  AFL: atrial flutter  Hb: hemoglobin  TP: total protein  Alb: albumin  Cre: creatinine  eGFR: estimated glomerular filtration rate  BUN: blood urea nitrogen  Na: sodium  K: potassium  Cl: chloride | | | |
